# Novel fabrication technique for high-resolution spherical crystal analyzers using a microporous aluminium base

**DOI:** 10.1107/S1600577522001886

**Published:** 2022-04-01

**Authors:** Ayman H. Said, Jung Ho Kim, Emily K. Aran, Thomas Gog

**Affiliations:** a Advanced Photon Source, Argonne National Laboratory, 9700 South Cass Avenue, Lemont, IL 60439, USA

**Keywords:** spherical crystal analyzers, microporous aluminium, inelastic X-ray scattering

## Abstract

Modern inelastic X-ray spectrometers employ curved, bent and diced analyzers to capture sufficiently large solid angles of radially emitted scattered radiation emanating from the sample. Fabricating these intricate analyzers, especially when a high energy resolution of a few millielectronvolts is required, is very time-consuming, expensive and often a hit-or-miss affair. A novel fabrication technique is introduced, utilizing a concave-spherical, microporous aluminium base to hold an assembly of a thin glass substrate with a diced crystal wafer bonded to it. Under uniform vacuum forces, the glass substrate is drawn into the aluminium base, achieving the desired bending radius, while dicing of the diffracting crystal layer prevents bending strain from being imposed on the individual crystal pixels. This technique eliminates the need for permanently bonding the crystal assembly to the concave lens, offering the opportunity for correcting figure errors, avoiding long-term degradation of the permanent bond, and making both lens and crystal reusable. Process and material costs are thus substantially decreased. Two analyzers, Si(844) and Ge(337) with intrinsic resolutions of 14.6 meV and 36.5 meV, respectively, were produced in this fashion and characterized in resonant inelastic X-ray scattering (RIXS) measurements. The achieved overall energy resolutions for both analyzers were 29.4 meV for Si(844) and 56.6 meV for Ge(337). Although the RIXS technique is veru sensitive to analyzer imperfections, the analyzers were found to be equal, if not superior, in quality to their traditional, permanently bonded counterparts.

## Abstract 2

A new fabrication technique for spherical crystal analyzers using a microporous aluminium base is introduced. It eliminates the need for permanent bonding of the crystal to the substrate, avoiding long-term degradation of the permanent bond and making the base and crystal reusable. Using this fabrication method, diced Si(844) and Ge(337) analyzers have been characterized with high-resolution resonant inelastic X-ray scattering, a technique which is particularly sensitive to analyzer imperfections.

## 1. Introduction

For X-ray scattering techniques, where radiation from a point source needs to be captured with a sufficiently large solid angle as well as appropriate energy resolution, spherical crystal analyzers in a near-backscattering Rowland circle geometry are most often used. Examples of such techniques include resonant (RIXS) and non-resonant inelastic X-ray scattering (IXS) (Huotari *et al.*, 2005 ▸; Shvyd’ko *et al.*, 2013 ▸), X-ray absorption spectroscopy (XAS) and X-ray emission spectroscopy (XES) (Seidler *et al.*, 2014 ▸), X-ray Raman spectroscopy (XRS) (Fister *et al.*, 2006 ▸; Sokaras *et al.*, 2012 ▸; Huotari *et al.*, 2017 ▸), and many others.

A spherical crystal analyzer typically consists of thin, bent crystal wafers mounted in a spherical substrate for lower-energy resolution in the electronvolt or sub-electronvolt regime (Collart *et al.*, 2005 ▸). However, when high-energy resolution below ∼100 meV is required, these analyzers need to be diced, creating an array of mm^2^-sized, unstrained, ideal flat crystal blocks arranged on a spherical surface. In either case, fabricating these complicated devices is an expensive, time-consuming and often hit-or-miss process; this is based on the authors’ experience and the feedback from other beamlines.

The theoretical energy resolution of a spherical analyzer is determined by fundamental design parameters, such as the energy, crystal material, reflection and geometrical contributions. In practice, however, factors related to the fabrication process, such as figure errors, strain induced in the crystal and long-term degradation of the spherical shape, can substantially diminish the energy resolution of the analyzer (Gog *et al.*, 2013 ▸).

**Figure 1:**
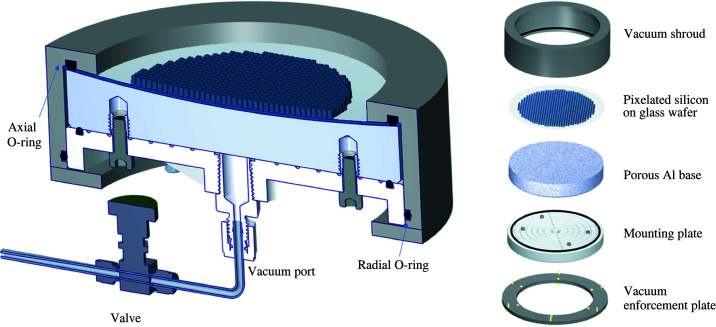
Schematic of the vacuum analyzer used in this work. On the left is a cross section view, on the right is an exploded view.

**Figure 2:**
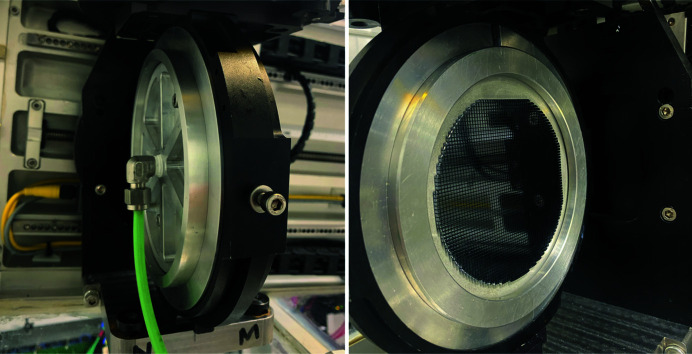
Front and back views of the analyzer mounted inside the spectrometer at sector 27 at the APS.

In this article, a novel production technique is introduced, which was successfully employed to fabricate diced, spherical crystal analyzers with very high energy resolution, suitable for the very demanding, high-resolution resonant inelastic X-ray scattering (RIXS) technique (Gog *et al.*, 2013 ▸; Said *et al.*, 2018 ▸). This technique utilizes a porous aluminium base with a precision-machined spherical surface. A thin crystal assembly, which includes a diced crystal wafer of a desired material and reflection on a glass substrate, is placed on the base and is drawn into the spherical form by uniform vacuum forces when the base is pumped through the mounting plate, as shown in Fig. 1 ▸. Two types of analyzers, employing Si(844) and Ge(337) reflections with intrinsic resolutions of 14.6 meV and 36.5 meV, respectively, were produced in this fashion and characterized with RIXS. This technique is particularly sensitive to analyzer imperfections like figure errors and strain. The new analyzers were found to be equal, if not superior, in quality to their traditional, permanently bonded counterparts, as shown in Fig. 2 ▸.

Traditionally, spherical crystal analyzers are manufactured by preparing a suitable thin crystal wafer and permanently mounting it in a spherical substrate through anodic bonding or gluing (Masciovecchio *et al.*, 1996 ▸; Said *et al.*, 2011 ▸; Ketenoglu, 2021 ▸; Verbeni *et al.*, 2005 ▸; Collart *et al.*, 2005 ▸; Macrander *et al.*, 1995 ▸). Since the last step is irreversible, any deficiency or long-term degradation of the bond will likely incur permanent figure errors and will render the analyzer obsolete. The described method allows for the reuse of the porous aluminium base with any crystal analyzer, and therefore eliminates the need for permanent concave lenses, thus reducing the overall cost of making spherical analyzers. Recently, a similar production technique has been reported for spherical and toroidal bent crystal analyzers with ∼1 eV resolution (Jahrman *et al.*, 2019 ▸), which eliminates the permanent bonding step. Instead, a thin, flexible, X-ray permeable membrane seals the space containing the thin crystal wafer and its curved substrate. When the vacuum is generated in this space, the membrane presses the crystal into the substrate, thus forming the desired curved surface.

## 2. Methods

A schematic view of the newly fabricated analyzer is shown in Fig. 1 ▸. The novel component of this assembly is a porous aluminium base, machined with one concave spherical surface of the desired radius and one plano surface. A diced crystal wafer of a desired material and reflection, providing an array of small crystalline pixels, is bonded to a glass (or silicon) substrate and placed on the base. The porous aluminium base is fastened on a mounting plate, which includes the vacuum port, and then placed inside a vacuum shroud. As shown in Fig. 1 ▸, an axial O-ring seals against the glass wafer from the top surface and a radial O-ring seals against the mounting plate from the lateral surface. By pumping the porous aluminium base through the mounting plate, vacuum levels between 10^−5^ mbar and 10^−6^ mbar can be routinely achieved. Under the uniform vacuum forces generated, the glass wafer is drawn into a near-perfect spherical shape as indicated by experimental measurements of the focal spot. While our experimental tests indicate that the spherical shape of the analyzer is already reliably attained at a vacuum level of 10^−1^–10^−2^ mbar, the vacuum seal design has proven to be leak-tight to the level of 10^−6^ mbar. This allows the analyzer to be pumped only initially while staying under sufficient vacuum for days or even a few weeks without further pumping and compromising the performance of the analyzer. The leak rate of the seal is even low enough to place the system in a helium atmosphere without detrimental effect on its operation.

Porous aluminium is used in industry for vacuum chucks that securely hold thin materials in grinding and polishing operations. The porous aluminium material for the spherical base of the new analyzer system was produced by Portec Ltd (Switzerland) and procured through NEST Technologies Inc. (http://www.nesttechnologies.com/), while Engis Corp. performed the precision spherical grinding. All other parts of the assembly were manufactured by the authors at the Advanced Photon Source (APS).

**Figure 3:**
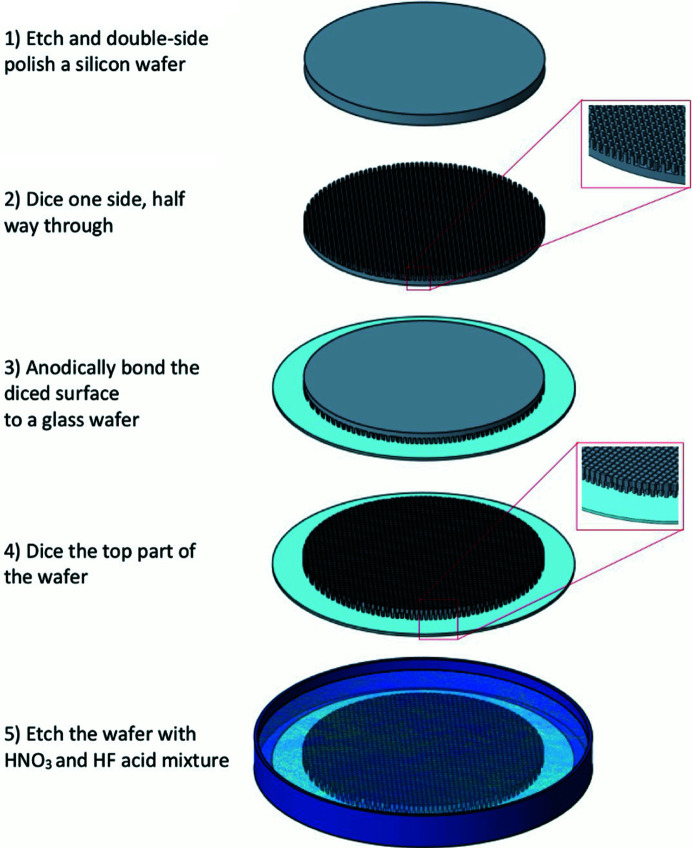
Schematic showing the fabrication steps for diced analyzers.

The pixelated crystal wafers were prepared at the APS as follows. (1) A 2 mm-thick double-sided polished crystal wafer with a 100 mm diameter serves as the starting material. (2) One side is diced about halfway through, using a 200–300 µm-thick dicing blade. The pixel size depends on the required energy resolution. (3) The diced surface is bonded to a 0.4–0.5 mm-thick glass wafer via anodic bonding or a proper adhesive. The bonding method depends on the crystal. The thickness of the glass depends on the bending radius needed. A thickness of 0.4–0.5 mm for the supporting wafer works for 1 m and 2 m radii. (4) The top surface of the wafer is diced using a 100 µm-thick blade. The cuts from the top should match the grooves from the bottom to have free-standing pixels. (5) The wafer is etched for 10 min at room temperature in a nitric acid (HNO_3_) and hydro­fluoric acid (HF) mixture (93:7 in volume, respectively). Etching requirements, including the type of solution, duration and temperature, depend on the crystal. A schematic of the fabrication steps is shown in Fig. 3 ▸.

## 3. Results

**Figure 4:**
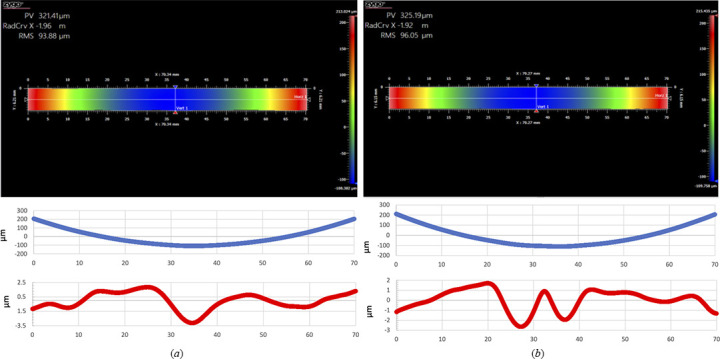
The radius of curvature of the aluminium base along two perpendicular directions on the base, (*a*) *X* and (*b*) *Y*. The blue symbols show the height profile in the measured area, and the red marks show the deviation of the measured radius from a sphere.

One of the undesired contributions to the energy resolution of an analyzer is its figure error. Therefore, it is crucial to achieve good focusing with the desired focus size, which is about twice the size of the analyzer pixel. The radius of curvature of the aluminium base was measured by visible-light optics in the metrology laboratory at the APS. This was carried out using a 0.5 mm glass wafer coated with a thin gold film and then mounted on the aluminium base under vacuum. The designed radius for the vacuum base is 2 m. The measurements were performed in two perpendicular directions on the aluminium base (*X* and *Y*). The measured area along each path was 70 mm × 6 mm, as shown in Fig. 4 ▸. For the measurements a NexView optical surface profiler was employed (https://www.zygo.com). The average measured radius was 1.94 m which is within the specifications of the aluminium base.

X-ray measurements were completed at the RIXS beamline at sector 27 at the APS. One of the first tests carried out is to image the beam diffracted by the analyzer at the detector. This was done using a pixelated detector (CdTe LAMBDA 60k 2D detector). The analyzers were fabricated from Si(844) for the Ir *L*_3_ absorption edge at *E* = 11.215 keV and Ge(337) for the Cu *K* absorption edge at *E* = 8.9808 keV. The incident beam generated by a diamond(111) high-heat-load monochromator is further monochromated by a high-resolution monochromator, 4× asymmetric Si(844) at *E* = 11.215 keV with a bandwidth of 15 meV and 4× asymmetric Si(440) at *E* = 8.908 keV with a bandwidth of 35 meV.

**Figure 5:**
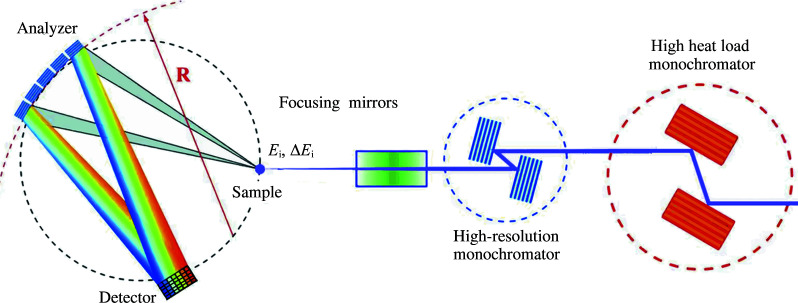
Schematic view of the RIXS spectrometer at beamline 27-ID at the APS.

A Kirkpatrick–Baez mirror system focuses the incident beam on the sample to a size of approximately 10 µm × 40 µm (v × h) at full width at half-maximum (FWHM). A 50 µm-thick Kapton tape was used as a sample for elastically scattered radiation, and the RIXS spectrometer arm was positioned at a 2θ scattering angle of 10°. The sample, spherical diced analyzer and detector were placed on a 2 m-diameter Rowland circle. A schematic of the RIXS spectrometer setup at beamline 27-ID at the APS is shown in Fig. 5 ▸ (Gog *et al.*, 2013 ▸).

The CdTe 2D detector has a pixel size of 55 µm × 55 µm. The vertical pixel size is larger than the required size to achieve the required energy resolution (Shvyd’ko *et al.*, 2013 ▸). In order to minimize the pixel size contribution to the total energy resolution, the detector was angled in the energy-dispersive direction, effectively giving rise to a pixel size of 25 µm × 55 µm. Tilting the detector did not introduce crosstalk between neighboring pixels. This was possible because the attenuation length of the CdTe is small (<15 µm) in the measured X-ray energy range in this work.

**Figure 6:**
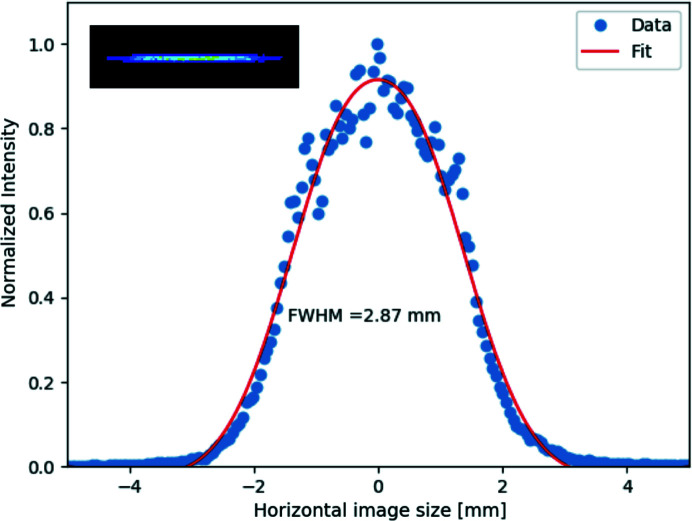
Horizontal beam intensity profile imaged by a 2D detector. The blue closed circles are the data, and the red line is the fit to the data. The inset is the actual image observed by the detector.

Fig. 6 ▸ shows the data and the fit to the horizontal intensity profile at the detector. In the inset, the intensity profile of the focused beam is shown. The FWHM of the image is 2.87 mm, which agrees well with the expected size of twice the pixel size of the analyzer.

A further test performed measured the overall energy resolution of the instrument. The energy resolution is the convolution of the energy resolution of the monochromator and the analyzer. As described above, a Kapton tape sample served as a scattering source. The energy of the analyzer was scanned at a fixed incoming beam energy from the monochromator. The energy scans were carried out by scanning the analyzer angle and the detector position to maintain the sample, analyzer and detector on the Rowland circle (Shvyd’ko *et al.*, 2013 ▸; Gog *et al.*, 2013 ▸).

**Figure 7:**
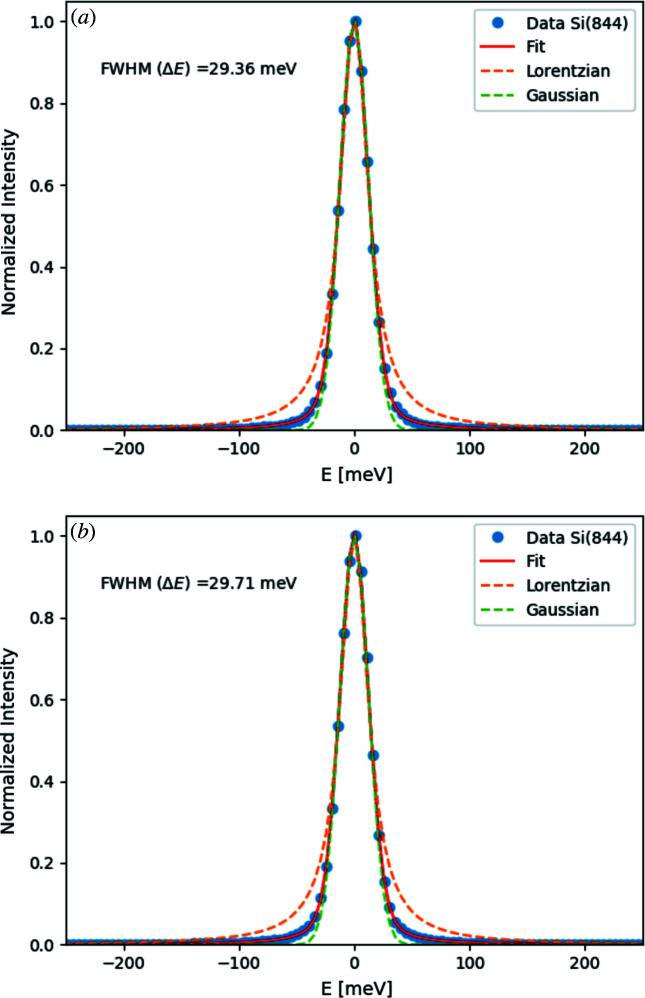
Measured energy resolution function of (*a*) the data taken with the vacuum-mounted analyzer and (*b*) the traditional glued analyzer. Closed circles correspond to the data; solid red lines show the pseudo-Voigt, dashed red lines show a Lorentzian function and green dashed lines indicate a Gaussian function.

**Figure 8:**
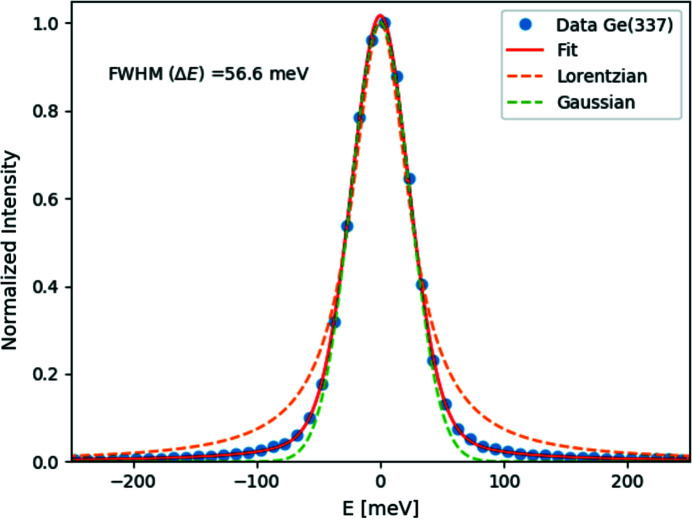
Measured energy resolution function from the Ge(337) analyzer. Closed circles correspond to the data; solid red lines show the pseudo-Voigt, dashed red lines show a Lorentzian function and green dashed lines indicate a Gaussian function.

Fig. 7 ▸ shows the measured resolution functions from Si(844) at *E* = 11.215 keV for the vacuum-mounted analyzer [Fig. 7 ▸(*a*)] and a traditional glued analyzer. The measured overall instrument resolution function from the vacuum-mounted analyzer is 29.4 meV and agrees very well with both the resolution function from a traditional glued analyzer and the theoretical predictions. This confirms that the new fabrication technique generates analyzers fully equal in quality to the traditional method, but with the added advantages mentioned above. Fig. 8 ▸ shows the energy resolution measured from the Ge(337) analyzer at *E* = 8.908 keV. The overall energy resolution from the Ge analyzer is 56.6 meV. The achieved energy resolution from the Ge(337) analyzer surpasses all previously made Ge(337) analyzers; for more information about the two analyzers used in this work, see Table 1 ▸.

## 4. Summary

In this work, we demonstrated a novel fabrication technique to produce high-energy-resolution analyzers for high-resolution inelastic scattering spectroscopy. The new method utilizes a microporous aluminium base holding a thin crystal wafer by uniform vacuum forces. This technique eliminates the need for permanently bonding the crystal assembly to the substrate, offering the opportunity for correcting figure errors, avoiding long-term degradation of the permanent bond, and making both substrate and crystal reusable. Process and material costs are thus substantially decreased. A microporous aluminium base can be used to make other types of analyzers, including cylindrical analyzers and bent analyzers for medium-energy resolution applications.

## Figures and Tables

**Table 1:** Summary of the parameters used in fabricating the two analyzers tested in this work

Energy (keV)	11.2150	8.9808
Analyzer crystal reflection	Si(844)	Ge(337)
Bragg angle (°)	85.77	87.14
Analyzer crystal intrinsic energy resolution (meV)	14.6	36.5
Incident energy resolution (meV)	15	35
Measured energy resolution (including the convolution of the intrinsic and geometrical contributions) (meV)	29.4	56.6
Pixel size (before etching) (mm × mm)	1.55 × 1.55	1.55 × 1.55
Analyzer illumination diameter opening (mm)	100	100
Bonding method between diced crystal and supporting wafer	Anodic bonding	Glue
Etching solution/etching time	10% HNO_3_ + 90% HF / 10 min	10% HNO_3_+ 90% HF / 10 min
